# Anesthetic approach to pregnant patients with malaria: a narrative review of the literature

**DOI:** 10.1186/s44158-024-00185-z

**Published:** 2024-07-26

**Authors:** Itay Zahavi, Meir Fons, Michal Meir, Mark Volevich, Emilia Guasch, Mark Nunnally, Sharon Einav

**Affiliations:** 1 The Bruce and Ruth Rappaport Faculty of Medicine, Technion–Israel Institute of Technology, Haifa, Israel; 2 The Faculty of Medicine of the Hebrew University, Jerusalem, Israel; 3 The Pediatric Infectious Disease Unit, Ruth Rappaport Children’s Hospital, Rambam Health Care Campus and Bruce and Ruth Rappaport Faculty of Medicine, Technion–Israel Institute of Technology, Haifa, Israel; 4 Anaesthesia and Reanimation Department, Hospital Universitario Fundación Jiménez Díaz, Madrid, Spain; 5 Departments of Anesthesia, Perioperative Care and Pain Medicine, Neurology, Surgery and Medicine, New York University, New York City, NY USA; 6 The Hebrew University Faculty of Medicine, Jerusalem, Israel; 7 Maccabi Healthcare Services, Sharon Region, Israel; 8 Medint Medical Intelligence Ltd, Tel-Aviv, Israel

**Keywords:** Pregnancy complications, Central nervous system infections, Malaria, Antimalarials, Anesthesia

## Abstract

**Introduction:**

Anesthesiologists play an important role in the management of labor and delivery during acute malaria infection. The peripartum anesthesia considerations for such cases remain unclear.

**Findings:**

Important peripartum considerations include the severity of thrombocytopenia and coagulopathy, hemodynamic status and cardiac disease, and the likelihood of central nervous system (CNS) involvement. Several antimalarial drugs may interact with perioperative medications, causing hypoglycemia, methemoglobinemia, or QT prolongation. Labor should usually not be induced. Patient volume status should be optimized pre-induction, but fluids should be administered with caution given the risk of cerebral edema. In case of CNS involvement intracranial pressure should be maintained. Case reports describe the successful use of neuraxial anesthesia but this approach requires further confirmation of safety. Despite the risks accompanying airway management in pregnancy, in some cases, general anesthesia was preferred due to the chance of CNS infection and disease complications. Tight postoperative assessments of neurological and bleeding status are indicated regardless of the mode of delivery.

**Conclusions:**

Despite the prevalence of malaria, the perioperative risk and preferred mode of anesthesia for pregnant patients with acute malaria remain under-researched and outcome data are limited.

## Background

**Fig. 1:**
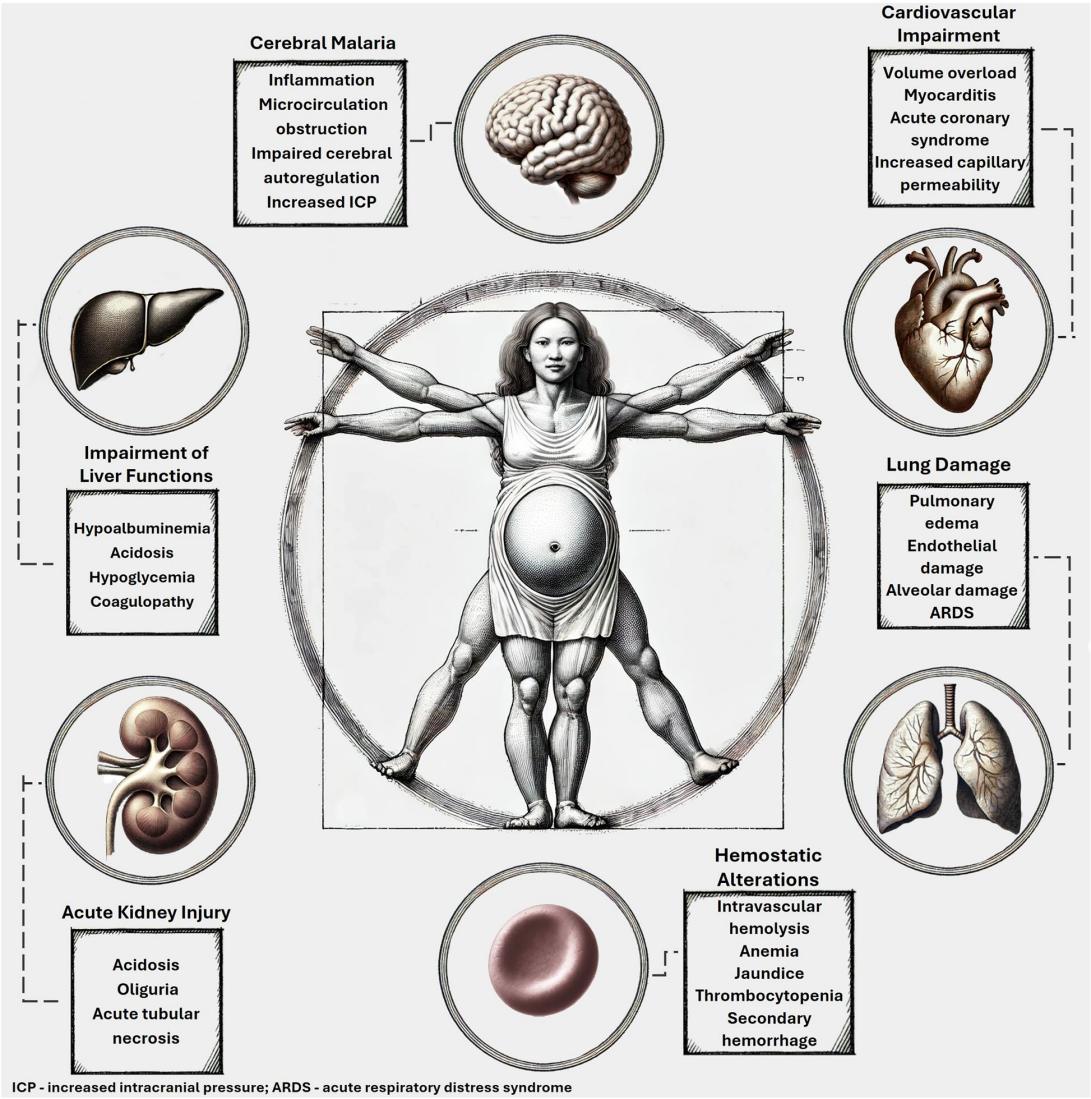
Clinical manifestations of severe malaria

Malaria, a disease caused by Plasmodium (P) species, accounts for approximately 250 million cases and 600 thousand deaths annually globally [1]. Severe malaria is usually associated with high parasite loads, and hyperparasitemia thresholds vary based on the species of Plasmodium and the immune status of the patient [2]. The clinical manifestations of severe disease include impairment of liver function (i.e., acidosis, hypoglycemia, coagulopathy), thrombocytopenia, severe hemolysis with resultant anemia and jaundice, renal failure (i.e., acidosis, oliguria, acute tubular necrosis), direct and indirect lung damage (i.e., endothelial and alveolar damage, ARDS), cardiovascular impairment, and shock (Fig. 1). Secondary hemorrhage, volume overload, and pulmonary edema are also described [3].

Pregnant women are three times more likely to have severe malaria than their nonpregnant counterparts and the mortality rate among pregnant women with severe malaria may approach 50% [4, 5]. Although two vaccines against Malaria have been available since 2021 (RTS, S/AS01, and R21/Matrix-M), these are currently recommended only for children [6]. Across the 38 countries in the WHO African region with moderate to high malaria transmission, an estimated 12.7 million pregnancies (one in three) were exposed to malaria infection in 2022 [1].

Most cases are probably asymptomatic [7]. Local mosquito-borne transmission of malaria has also been recently identified in Florida, Texas, and Maryland which are non-endemic [8, 9]. In the UK, the overall number of imported malaria cases was 1369 in 2022 and 2,093 in 2023 [10]. In Italy, the number of malaria cases per 100,000 population was 571 in 2022 [11].

Cerebral malaria predominantly affects adults with immune suppression, children, and pregnant women [12, 13]. This potentially lethal complication, mostly of *P. falciparum* infection, stems from local inflammation, obstruction of the cerebral microcirculation, and impaired cerebral autoregulation, causing an increase in intracranial pressure and malignant brain edema [14]. Symptoms include impaired consciousness, seizures, and ultimately death [13] (Fig. 1).

We aimed to outline the considerations that may be relevant to anaesthesiologists treating a pregnant woman with malaria during labor and delivery.

## Method

We searched PubMed, Google Scholar, and the Cochrane databases for any type of paper reporting original data regarding the mode of anesthesia and outcomes in hospitals for pregnant women with acute malaria undergoing labor and delivery. The search was conducted twice (up to 27th May 2024), each time by a single researcher (MF and IZ) to ensure thoroughness. The search terms and results are presented in Appendix 1. The reference lists of relevant articles were also manually scrutinized for additional potentially pertinent articles by all authors. No language restriction was applied during the search but only papers in English were screened. Potentially relevant papers were identified through title and abstract and downloaded in full for information extraction by two of the authors (MF and IZ). The information from these papers was then gathered conjunctly into a narrative summary of the literature by the two authors (MF and IZ). This content was reviewed and adjusted to best reflect the existing information by a third party (SE).

## Discussion

### The pathophysiology of malaria during pregnancy

The degree of adherence of infected erythrocytes to the placenta suggests the likelihood of severe malaria and poor pregnancy outcomes. *P. falciparum* and *P. Knowlesi* are more commonly associated with severe maternal–fetal outcomes [15, 16]. *P. Vivax, P. Ovale*, and *P. Malaria* infection infrequently results in severe outcomes [16, 17].

Successful pregnancy outcome requires the maintenance of a delicate balance between proimmune and anti-immune influences that allow placental development and fetal tolerance [18]. Many of the regulatory and cellular processes underlying these changes have yet to be elucidated. During malaria, massive mononuclear intervillous inflammatory infiltration has been associated with lower birth weights. Perivillous fibrin depositions have been associated with an increased risk of premature delivery [19]. Hence the time available for pre-delivery preparations may also be briefer than planned. The risk of transplacental malaria transmission is 1.5% in immune pregnant women and 7–10% in semi-immune and nonimmune women [20, 21]. All malaria species may cause congenital disease.

### Malaria severity and labor considerations

Uncomplicated malaria is characterized by fewer than 2% parasitized erythrocytes without signs of severity or complicating features. Although any patient diagnosed with malaria during pregnancy should be admitted to the hospital due to the possibility of rapid deterioration, current guidelines suggest that labor should not be induced in uncomplicated malaria (Grade A) [22]. This suggestion is based on data from several prospective studies including 3000 pregnant women with uncomplicated malaria that were followed from diagnosis of malaria through treatment and birth. However, while the majority of those studies [23, 24] include data about outcomes such as premature labor and stillbirth, these papers actually present no data on labor induction.

Labor management should also follow protocol in case of fetal or maternal distress, including the timely use of interventions (e.g., forceps, vacuum, surgery) [22].

Severe malaria requires multidisciplinary care due to the combined risk of premature labor and maternal deterioration [25]. Even in severe malaria labour should only be induced for obstetric indications. If the patient is hemodynamically unstable, complete blood counts and coagulation profiles should assist in diagnosing occult hemorrhage. Dynamic evaluation of intravascular volume status should always precede fluid administration. A multicentre randomized controlled trial described increased 48-h mortality rates in low-resource settings among children receiving fluid resuscitation for treatment of septic shock, regardless of the type of fluid administered. After malaria was confirmed in 57% of the children, the increased mortality was attributed to the worsening of cerebral edema in response to fluid administration [26]. To date no equivalent study has been conducted in adults with malaria, therefore fluids should be administered judiciously.

A systematic review identified hypoalbuminemia in patients with malaria as an important indicator of severe disease, and in the general population, hypoalbuminemia is often due to liver and kidney failure as well as increased capillary permeability [27]. Additionally, in endemic regions, more than 40% of pregnant women may have hypoalbuminemia at their first antenatal visit [28]. A systematic review of malaria in the perioperative setting highlighted the risk of iatrogenic fluid overload resulting in pulmonary edema in the presence of hypoalbuminemia and low oncotic pressure [29]. Restrictive fluid administration has been associated with renal failure in hemodynamically compromised critically ill patients. While there is an increasing rate of peripartum renal failure, our search yielded no case reports ascribing renal failure to intravascular depletion in malaria.

### Selecting the mode of anesthesia

*Neuraxial anesthesia i*s usually preferred over general anesthesia as the risks of airway management during pregnancy may be exacerbated with malaria by soft tissue edema. Furthermore, despite concerns relating to parasite transfer into the Cerebrospinal fluid (CSF) through spinal anesthesia, no such case has been reported [29]. Plasmodium species cannot replicate in the CSF and are confined to cerebral capillaries [29]. Acute malaria may cause coagulopathy and thrombocytopenia during pregnancy [30]. It is therefore prudent to ensure that coagulation is unimpaired and platelet levels exceed the threshold for neuraxial anesthesia (50,000 to 80,000 10^6/L) (Table 1).

**Table 1:** Guideline recommendations for neuraxial procedures in thrombocytopenic patients

Society	Recommendation
The Society for Obstetric Anesthesia and Perinatology (SOAP) 2021 [31]	Avoid neuraxial procedures if platelets < 50,000 × 10^6/L (Strength of recommendation 2B, Level of evidence C-LD)
The Association of Anaesthetists of Great Britain and Ireland (AAGBI) 2013 [32]	Obstetric patients with ITP and a platelet count of 20,000–50,000 × 10^6/L are at high risk for spinal hematoma
The British Society for Haematology 2015 [33]	Advise against epidural anesthesia for obstetric patients with Acute Myeloid Leukemia if the platelet count is less than 80,000 × 10^6/L. (Strength of recommendation 1C)
The Sociedade Brasileira de Anestesiologia (SBA) 2014 [34]	Epidural or spinal blocks, in the absence of risk factors for bleeding, may be performed with platelet counts greater than 80,000 × 10^6/L (Level of evidence D)
The French Safety Agency for Health Products (AFSSaPS) 2003 [35]	A platelet count > 50,000 × 10^6/L suffices for spinal anesthesia
	A count greater than 80,000 × 10^6/L suffices for epidural anesthesia
	Consider other risk factors as well as the progressive nature of thrombocytopenia

Barring evidence of coagulopathy, cerebral malaria, or hemodynamic compromise, performing neuraxial anesthesia is probably reasonable in most cases. However, more research is clearly needed regarding the safety of neuraxial anesthesia as our search identified only case reports (Table 2).

**Table 2:** Cases with information on neuraxial or general anesthesia during delivery among women with acute malaria

Case report	Mode of anesthesia	Urgency of caesarean delivery	Reason reported for selection of mode of anesthesia	Drugs used	Outcomes
Zanfini et al. 2016 [36]	Neuraxial (spinal)	Urgent	Absence of coagulopathy, thrombocytopenia, secondary bacterial sepsis, or cerebral malaria with elevated ICP	*Intrathecal:* Hyperbaric bupivacaine 0.5% 10 mg Sufentanil 5 µg	Stable blood pressure, successful postoperative analgesia, decreased maternal parasitemia, complete symptom resolution, neonate required oxygen support
Sultan et al. 2012 [37]	Neuraxial (spinal)	Elective	Advantages of neuraxial anesthesia over general, mild anemia, thrombocytopenia, and elevated liver transaminases not observed during pregnancy	Premedication: *Intravenous:* Metoclopramide 10 mg Ranitidine 50 mg Hydrocortisone 100 mg Cefazolin 1 g Induction and maintenance: *Intrathecal:* Hyperbaric bupivacaine 0.75% 12 mg Fentanyl 10 µg Morphine 200 µg Post-delivery: *Intravenous:* Phenylephrine 1 mg Fentanyl 150 µg	Severe complication of coronary artery dissection postoperatively, but causality with babesiosis is ambiguous
Mathew et al. 2011 [38]	General	Urgent	Hemodynamic instability, declining platelet count, systemic sepsis, urgency of the situation, and absence of immediate coagulation profile	Premedication: *Oral* Sodium citrate 30 mL *Intramuscular* Betamethasone 12 mg *Intravenous* Metoclopramide 10 mg Ranitidine 50 mg Induction and maintenance: *Intravenous* Thiopental and suxamethonium in rapid-sequence technique, unknown dose *Inhaled* Nitrous oxide isoflurane Post-delivery: *Intravenous* Oxytocin 5 U IV bolus, 40 U/500 mL saline drip Morphine 10 mg Co-amoxiclav 1.2 g Gentamicin 240 mg Post-surgery: *Intravenous* Quinine 20 mg/kg loading dose, further doses of 10 mg/kg every 8 h Cefotaxime unknown dose *Oral* Primaquine 15 mg daily for 14 days	Rapid post-surgery improvement, no infection or malaria in the placenta, normalized platelet count, baby recovered well
Samanta et al. 2014 [39]	General	Urgent	Thrombocytopenia, coagulopathy, suspected raised ICP, impaired mental status, risk of introducing malarial parasites into CSF with regional anesthesia	Induction and maintenance: Fentanyl unknown dose Atracurium unknown dose *Inhaled* Isoflurane Post-delivery: Noradrenaline unknown dose Transverse abdominis plane block for pain	Postoperative transfer to ICU, pulmonary hemorrhage, and jaundice managed, significant post-operative improvement

*ICP* Increased intracranial pressure, *CSF* Cerebrospinal fluid, *mg* milligram, *ICU* Intensive care unit, *mL* Milliliter, *kg* Kilogram

^*^Babesiosis is caused by *Babesia microti* parasites. It shares pathobiological and clinical features with *falciparum malaria*, such as a high fever, anemia, dark urine, jaundice, and kidney failure [40]

*General anesthesia* has also been described in pregnant patients with acute malaria (Table 2). Other than airway and hemodynamic management, maintenance of cerebral perfusion and intracranial pressure may require attention. During normal pregnancy, cerebral blood flow increases but intracranial pressure remains unaltered. Disruption of normal protective mechanisms may, however, increase the risk of cerebral edema. Furthermore, the first and second stages of labor may be accompanied by severe increases in intracranial pressure (39 mmHg and 71 mmHg, respectively) [41]. During normal pregnancy, blood–brain barrier permeability remains largely unchanged. Increased blood–brain barrier permeability is seen in animal models of preeclampsia, but whether this occurs in humans remains unknown. There is also weak evidence that Aquaporin 4 overexpression may influence brain edema formation and resolution in the second half of gestation [42]. Our search identified no descriptions of anesthesia management in pregnant women with CNS involvement in acute malaria.

### Management of general anesthesia

Premedication with a benzodiazepine may be useful for both anxiolysis and seizure prevention. For malaria patients, the use of diazepam is most commonly described [43].

Patients with acute malaria may have low baseline PaCO2 levels [29]. Efforts employed to maintain intracranial pressure during induction and intubation should therefore include prevention of transient peri-intubation hypercapnia. In addition, blunting of the response to laryngoscopy and intubation with local anesthetics and effective neuromuscular blockade to prevent coughing may be employed. During surgery, a 15° head-up position and avoiding the use of endotracheal tube ties may improve cerebral venous drainage [29]. Whether mannitol effectively manages intracranial pressure in cerebral malaria remains debatable. Therefore, mannitol should only be used for salvage during impending cerebral herniation [29].

Several antimalarial drugs may interact with drugs used during and after the perioperative period. Quinine may enhance neuromuscular blockade and may aggravate hypoglycemia [44]. Chloroquine reduces the effect of neostigmine and pyridostigmine. Mefloquine interacts with anticholinergic drugs (e.g., physostigmine) to produce central anticholinergic syndrome [45]. Dapsone (which has largely been abandoned), can cause methemoglobinemia [46].

A systematic review of cardiovascular complications in patients with symptomatic, usually severe, malaria (43 studies, 3,117 adults and children), found a pooled prevalence estimate of 7% (95% CI 5–9) for any cardiovascular complication. Cardiovascular pathologies included myocarditis and acute coronary syndrome. All histopathological studies identified parasitized erythrocytes in the myocardium [47]. Quinine and artemisinin-based combination therapies, first-line treatments for malaria in many malaria-endemic areas, have been implicated in QT prolongation [48, 49]. One case report has attributed new onset Brugada Syndrome in a patient with malaria to propofol [50]. In cases with cardiac involvement, ketamine should probably not be used for induction of anesthesia as it may affect cardiac conduction, cause arrhythmias [51], and increase cerebral blood flow and intracranial pressure [29].

All volatile anesthetics induce a dose-dependent increase in cerebral blood flow and reduce cerebral oxygen consumption. If immediate post-surgery tracheal extubation and neurological assessment are needed, the rapid offset and recovery from sevoflurane and desflurane may make these drugs more suitable than isoflurane for maintenance of anesthesia [29]. Since postoperative extubation is usually planned, sedative agents and opioids with a prolonged effect should be administered in measured doses if at all.

### Management after delivery

Post-delivery, the parturient should be managed in a highly monitored environment where disease exacerbation or complications may be rapidly identified and treated. Periodic neurological evaluation is recommended, particularly in cases with suspected central nervous system involvement. A retrospective analysis comparing women in malaria-endemic areas (*n* = 4023) to women in non-endemic areas (*n* = 6655) showed no increased risk of postpartum hemorrhage in women with malaria. However, among those with hemorrhage, a higher mean peripartum blood loss was observed [52]. A longitudinal study that followed women from antenatal care to the immediate postpartum period (*n* = 675) noted higher blood loss in the two hours after delivery among bleeding women with malaria compared to those without malaria. However, the method used to assess blood loss was inexact [16].

## Conclusions

Given the prevalence of malaria, our search (Appendix 1) yielded a surprising paucity of literature. Information on the management of anesthesia in pregnant patients with acute disease remains scarce. Much of our understanding is based on case studies, predominantly from regions where health infrastructure and resource limitations may affect management.

## Data Availability

No datasets were generated or analysed during the current study.

## Appendix

### Search terms (date of last search 27th May 2024)

“malaria” and “epidural” and “pregnancy”—0 results.

“malaria” and “spinal” and “pregnancy”—6 results, 2 relevant case reports.

Soltanifar D, Jacobs M, Jones T, McGlennan A, Sultan P. Spinal anaesthesia for emergency caesarean delivery in a parturient with falciparum malaria. Int J Obstet Anesth. 2015 Feb;24(1):91. 10.1016/j.ijoa.2014.07.003. Epub 2014 Jul 16. PMID: 25433574.

Zanfini BA, Dell'Anna AM, Catarci S, Frassanito L, Vagnoni S, Draisci G. Anesthetic management of urgent cesarean delivery in a parturient with acute malaria infection: a case report. Korean J Anesthesiol. 2016 Apr;69(2):193–6. 10.4097/kjae.2016.69.2.193. Epub 2016 Mar 30. PMID: 27066212; PMCID: PMC4823419.

“malaria” and “neuraxial” and “pregnancy”—0 results.

“malaria” and “neuraxial anesthesia”—1 relevant result (cited in the article).

(Soltanifar D, Carvalho B, Sultan P. Perioperative considerations of the patient with malaria. Can J Anaesth. 2015 Mar;62(3):304–18. 10.1007/s12630-014-0286-7. Epub 2014 Dec 4. PMID: 25471683; PMCID: PMC7102007.)

“intravascular depletion” and “malaria”—1 relevant result (cited in the article).

Pugliese CM, Adegbite BR, Edoa JR, Mombo-Ngoma G, Obone-Atome FA, Heuvelings CC, Bélard S, Kalkman LC, Leopold SJ, Hänscheid T, Adegnika AA, Huson MA, Grobusch MP. Point-of-care ultrasound to assess volume status and pulmonary oedema in malaria patients. Infection. 2022 Feb;50(1):65–82. 10.1007/s15010-021-01637-2. Epub 2021 Jun 10. PMID: 34110570; PMCID: PMC8803774.

"Babesiosis" + "spinal" + "pregnancy"- 1 relevant result (cited in the article).

Sultan P, Green C, Riley E, Carvalho B. Spinal anaesthesia for caesarean delivery in a parturient with babesiosis and Lyme disease. Anaesthesia. 2012 Feb;67(2):180–3. 10.1111/j.1365-2044.2011.06941.x. PMID: 22251109.

### Added search based on reviewers comments (16 July 2024)

“hypoalbuminemia” and “pregnancy” and “malaria”—no relevant results.

respiratory depression and diazepam and delivery—11 papers, 1 relevant (Chinese).

diazepam and placenta and respiratory depression—2 papers, none relevant.

benzodiazepine and placenta and respiratory depression—2 papers, none relevant.

"APGAR" and "diazepam" and "delivery" and "anxiolysis"—no relevant results.

"APGAR" and "diazepam" and "anxiolysis"—no relevant results.

"APGAR" and "diazepam" and "anesthesia"—24 papers, 1 relevant.

## Abbreviations

ICP: Increased intracranial pressure
CSF: Cerebrospinal fluid
ICU: Intensive care unit
P: Plasmodium
CNS: Central nervous system
ARDS: Acute respiratory distress syndrome
