# Correction: Anesthetic approach to pregnant patients with malaria: a narrative review of the literature

**DOI:** 10.1186/s44158-024-00189-9

**Published:** 2024-08-01

**Authors:** Itay Zahavi, Meir Fons, Michal Meir, Mark Volevich, Emilia Guasch, Mark Nunnally, Sharon Einav

**Affiliations:** 1 The Bruce and Ruth Rappaport Faculty of Medicine, Technion–Israel Institute of Technology, Haifa, Israel; 2 The Faculty of Medicine of the Hebrew University, Jerusalem, Israel; 3 The Pediatric Infectious Disease Unit, Ruth Rappaport Children’s Hospital, Rambam Health Care Campus and Bruce and Ruth Rappaport Faculty of Medicine, Technion–Israel Institute of Technology, Haifa, Israel; 4 Anaesthesia and Reanimation Department, Hospital Universitario Fundación Jiménez Díaz, Madrid, Spain; 5 Departments of Anesthesia, Surgery and Medicine, Perioperative Care and Pain Medicine, New York University, NeurologyNew York City, NY USA; 6 Faculty of Medicine, The Hebrew University, Jerusalem, Israel; 7 Maccabi Healthcare Services, Ra‘anana, Sharon Region Israel; 8 Medint Medical Intelligence Ltd, Tel-Aviv, Israel

**Correction: J Anesth Analg Crit Care 4, 48 (2024)**

**https://doi.org/10.1186/s44158-024-00185-z**

The original publication of this article contained an incorrect author name. The incorrect and correct information is listed in this correction article, the original article has been updated.

Incorrect

Micahl Meir

Correct

Michal Meir
